# Fractal Analysis of Lung Structure in Chronic Obstructive Pulmonary Disease

**DOI:** 10.3389/fphys.2020.603197

**Published:** 2020-12-21

**Authors:** Naoya Tanabe, Susumu Sato, Béla Suki, Toyohiro Hirai

**Affiliations:** ^1^Department of Respiratory Medicine, Graduate School of Medicine, Kyoto University, Kyoto, Japan; ^2^Department of Biomedical Engineering, Boston University, Boston, MA, United States

**Keywords:** chronic obstructive pulmonary disease, emphysema and chronic obstructive airways disease, computed tomogaphy, fractal, power law, airway - obstruction, histology, simulation - computers

## Abstract

Chest CT is often used for localizing and quantitating pathologies associated with chronic obstructive pulmonary disease (COPD). While simple measurements of areas and volumes of emphysema and airway structure are common, these methods do not capture the structural complexity of the COPD lung. Since the concept of fractals has been successfully applied to evaluate complexity of the lung, this review is aimed at describing the fractal properties of airway disease, emphysema, and vascular abnormalities in COPD. An object forms a fractal if it exhibits the property of self-similarity at different length scales of evaluations. This fractal property is governed by power-law functions characterized by the fractal dimension (FD). Power-laws can also manifest in other statistical descriptors of structure such as the size distribution of emphysema clusters characterized by the power-law exponent D. Although D is not the same as FD of emphysematous clusters, it is a useful index to characterize the spatial pattern of disease progression and predict clinical outcomes in patients with COPD. The FD of the airway tree shape and the D of the size distribution of airway branches have been proposed indexes of structural assessment and clinical predictions. Simulations are also useful to understand the mechanism of disease progression. Therefore, the power-law and fractal analysis of the parenchyma and airways, especially when combined with computer simulations, could lead to a better understanding of the structural alterations during the progression of COPD and help identify subjects at a high risk of severe COPD.

## Introduction

Chronic obstructive pulmonary disease (COPD) is a major respiratory disease that imposes a high social health burden worldwide (Adeloye et al., 2015). Airflow limitation measured by spirometry is a key physiological feature and gold standard to diagnose COPD. Emphysema and airway disease are two main pathological changes that contribute to airflow limitation, but their relative contributions to the disease differ among patients. Additionally, the heterogeneous spatial distribution of these two pathological changes in each patient complicates understanding of the structural basis of the disease. For example, COPD patients with similar airflow limitation may have substantial near homogeneously-distributed emphysema without airway disease, moderate upper-dominant heterogeneously-distributed emphysema with moderate airway disease, or little emphysema with severe airway disease (Schroeder et al., 2013; Lynch et al., 2015). Furthermore, morphological alteration of vessels is also a pathological feature, and pulmonary hypertension is a common comorbidity in patients with COPD (Matsuoka et al., 2010; Coste et al., 2019). A combination of these airway, lung parenchyma, and vessel lesions underlies the structural heterogeneity, which not only influences lung function but can be a driving factor for the disease progression in patients with COPD (Mondoñedo et al., 2019).

Histology is a gold standard for the morphological analysis of COPD lungs, but histology is too invasive to perform in live patients. Even when histological samples are obtained from a biopsy specimen, these samples are usually small or affected by other diseases, such as malignancy, and cannot represent the heterogeneous structural changes in whole lungs. Alternatively, CT is commonly used to estimate the pathology of the lungs and extrapulmonary abnormalities. CT is accessible and less invasive and allows for a 3D separate evaluation of airway disease, emphysema, and vascular abnormality for the whole lungs (Lynch and Al-Qaisi, 2013; Labaki et al., 2017). Indeed, the area and volume of emphysematous regions, the wall and lumen cross-sectional area (CSA) of airways, and the volume of smaller vessels have been measured and utilized as indexes of emphysema severity, airway remodeling, and reduced vascularity and shown to be associated with various outcomes of COPD (Grydeland et al., 2010; Haruna et al., 2010; Matsuoka et al., 2010; Han et al., 2011). Nonetheless, these simple quantifications of lung structure do not capture the complexity of the size and spatial distributions of the pathological features in COPD lungs.

Since the concept of fractals has often been applied in many scientific studies to evaluate complex phenomena and objects including lung morphology, this review is aimed at summarizing the fractal properties in nature and to describe the potential of the fractal concept for quantifying the complexity of airway disease, emphysema, and vascular abnormalities in COPD.

## What are Power Laws and Fractals?

**Figure 1:**
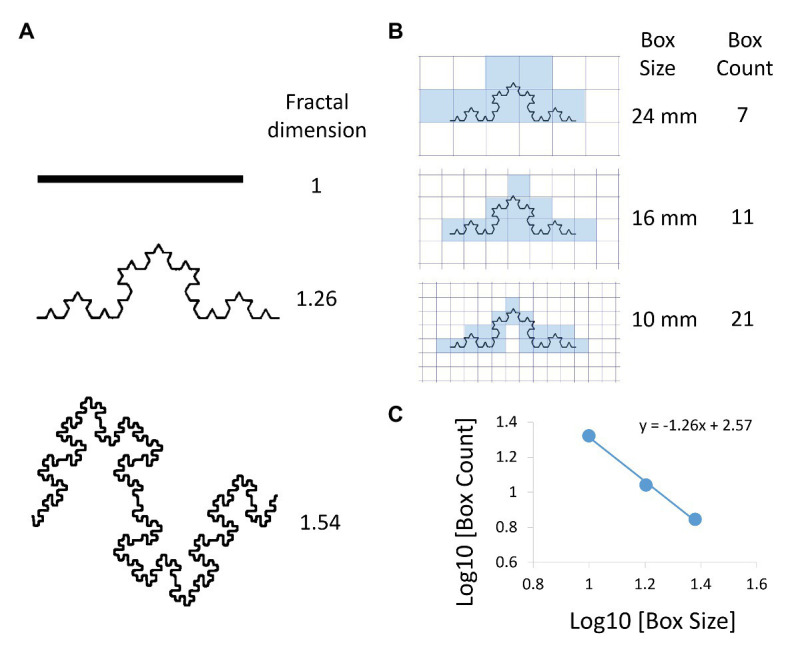
Examples of fractal objects and the box-counting method to determine their fractal dimension (FD). **(A)** Comparisons of FDs between three objects. **(B)** Box-counting method to calculate FD. A box grid at a given size scale x is superimposed on the object. The number of boxes that include a part of the object *N(x)* is counted. The analysis is repeated for different values of *x*. **(C)** *x* and *N(x)* are plotted on a double logarithmic scale and the FD is calculated as the negative slope of the regression line (FD = 1.26 in this case).

The concept of fractal geometry was introduced by Mandelbrot to evaluate the complexity of natural forms (Mandelbrot, 1977). An object forms a fractal if it exhibits the property of self-similarity at different length scales of evaluations, also called scale-free behavior (Figure 1A). This fractal property is governed by power law functions characterized by a single number, the exponent from which a non-integer dimension of the object, termed the fractal dimension (FD), can be calculated (Figure 1B; Suki et al., 2011). Among several methods to calculate the FD, such as box-counting, lacunarity, and mass-radius methods, the box-counting algorithm is the most commonly used (Figure 1C). A grid of boxes of size L is placed over the object, and the number of boxes that are needed to cover the objects [*N(n)*] is counted. If the relationship between *N(n)* and *L(n)* appears linear on a log-log plot, the data can be fitted with the straight line which represents a power-law functional form as *N(n)* = *k* × *L(n)*^−*D*^. The negative slope of the linear regression line corresponds to the FD that characterizes the complexity and space-filling capacity of the object. Regarding the lung structure, airway and vascular trees, as well as the alveolar surface, display fractal properties (Weibel, 1991, 2009). At first, researchers used histological sections and bronchial casts to calculate the FD in very few samples and also investigated the fractal geometry of the vascular tree in relation to the blood flow (Glenny and Robertson, 1990; Caruthers and Harris, 1994). Power laws can also manifest in other statistical descriptors of structure. If the measurement of the sizes of many objects, such as low attenuation area (LAA) clusters (see below), show that the histogram or distribution of sizes vary linearly with size on a log-log plot, then the distribution is said to follow a power law characterized by the exponent, or negative slope, D.

In the last decade, the technical advances in CT imaging and analysis software have made it possible to invoke fractal analysis applied to emphysema and airway disease in large populations and showed potential for FD and D as imaging biomarkers to predict COPD outcomes (Mishima et al., 1999; Coxson et al., 2008; Tanabe et al., 2011, 2018a; Tobino et al., 2017; Hwang et al., 2019; Shimizu et al., 2020). Unfortunately, despite much progress, the power-law and fractal analyses are less commonly used by clinicians and researchers compared to the simple quantitation of emphysema and airway disease. In addition to unfamiliarity with the advantages of the power-law and fractal analyses, the lack of standardized methods to obtain FD and D to characterize emphysema, airway, and vascular structures hinders the general application of the technology in clinical situations. Our hope is that summarizing the studies that used the power-law and fractal analyses for a better understanding of lung structure will help overcome the current limitations and facilitate the clinical applicability of these analysis methods. Moreover, this review provides directions of the application of the power-law and fractal analyses to the morphological evaluation of emphysema, airway disease, and pulmonary vascular disease in the management of COPD.

## Emphysema

The alveolar surface is the main site for gas exchange in respiration. The alveolar surface area in healthy persons is substantial (130 m^2^) for the chest cavity size (5–6 L), which facilitates gas exchange. To keep all alveoli open and connected to the atmosphere, the parenchyma shows a fine foam-like structure that is characterized by fractal properties (Weibel, 1991).

### Histology

**Figure 2:**
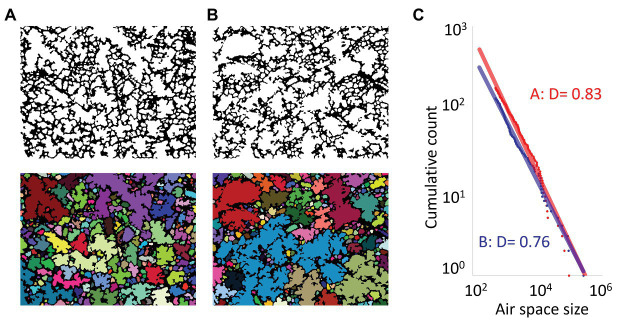
The power-law exponent D of the size distribution of airspaces to evaluate emphysema on lung tissue. Histological sections on digital images were binarized and color segmented for each airspace in control **(A)** and an emphysematous mouse **(B)**. **(C)** Size of each airspace was measured. Then, the cumulative count of airspaces larger than a given size was plotted on a double logarithmic scale, and the power-law exponent D was calculated as the negative slope of the regression lines (D = 0.83 and 0.76 for control and emphysematous mice, respectively). Of note, the power-law exponent D for the size distribution of airspaces is not the FD of airspace shape.

Sato et al. (2007) reported the presence of two power-law relationships describing the structure of the alveoli on histologic sections using the klotho mouse that spontaneously develops emphysema. The authors first calculated the perimeters and the areas of individual airspaces from histology and found a power-law relationship between them. The second power-law relationship, shown in Figure 2, was established for the size distribution of airspaces revealing that the airspace areas and their cumulative frequency can be plotted on a straight line on the log-log plot, and the negative slope of the fit defines the exponent D. Importantly, although the exponent D of the power-law for the size distribution of airspaces is not exactly the same as the FD of the alveolar shape, both reflect some aspects of the complexity of lung structure. This is in line with a previous work by Crawford et al. (1993) who investigated soil structure using size distribution of soil particles. The authors compared the particle-size distribution with the FD of particle structure and found that a power-law relationship for the particle size distribution does not necessarily indicate that the structure itself is fractal (Crawford et al., 1993).

Interestingly, the exponent D more sensitively reflects structural alterations in emphysematous lungs. The exponent D decreases even when the mean linear intercept, a standard histological index of airspace enlargement corresponding to emphysema severity, is unchanged (Sato et al., 2007). In contrast, another study calculated the FD of alveoli using the 3D box-counting method and found that elastase-induced emphysema in mice is characterized by a decrease in the FD, as well as an increase in mean linear intercept (Andersen et al., 2012). These differing conclusions are likely related to differences in the type and extent of emphysema between the two studies (Tanabe et al., 2011; Hwang et al., 2019). Nevertheless, these findings suggest that adding the power-law and fractal analyses to the standard quantification of airspace enlargement on histology can provide additional information on emphysematous lung structure.

### Computed Tomography

Quantitative CT measurements of emphysema have been intensively used in COPD research. There are two major emphysema indices. The first index is the percentage of the low attenuation area (LAA), which is defined as a region where the CT density is below a fixed threshold, such as −950 Hounsfield units (HU), relative to the total lung area [the percentage of LAA to the total lung area (LAA%) or the percentage of low attenuation volume to the total lung volume (LAV%); Madani et al., 2006; Parr et al., 2008]. The second index is the percentile point defined as the cut-off value in the HU below which a given percentage of all voxels, such as the 15th percentile, is distributed (Parr et al., 2006). Both of these indices reflect the extent of pathological emphysema on histologic slides (Gould et al., 1988; Muller et al., 1988; Gevenois et al., 1996). While these indices are unable to account for the size distribution of emphysema regions throughout the lung, clinical studies have made it possible to show that LAA or LAV% is associated with the loss of pulmonary function, symptoms, a reduction in the quality of life, lung cancer development, an increased future risk of exacerbations, rapid disease progression, and increased mortality in patients with COPD (Wilson et al., 2008; Haruna et al., 2010; Han et al., 2011; Vestbo et al., 2011; Nishimura et al., 2012).

**Figure 3:**
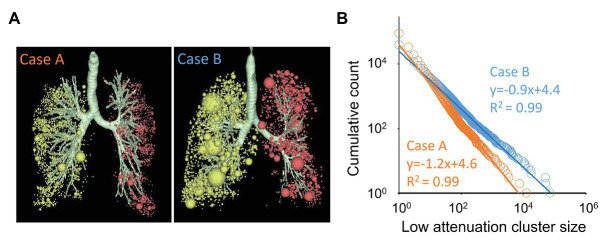
The power-law exponent D for the size distribution of emphysematous clusters on CT. **(A)** 3D visualization of low attenuation clusters (LACs) for two cases. Each sphere indicates a LAC that was identified as neighboring voxels in 3D on CT. Of note, many small clusters were found in case A, whereas several larger clusters and many small clusters were found in case B. **(B)** Cumulative count of LACs larger than a given size was plotted on a double logarithmic scale, and the power-law exponent D was calculated as the negative slope of the regression lines. The exponent D for cases A and B were 1.2 and 0.9, respectively.

The pioneer work by Mishima et al. (1999) introduced the size distribution of emphysematous regions by applying the concept of fractals in quantitative assessment of emphysema on CT. The study identified neighboring LAA pixels as a low attenuation cluster (LAC) and demonstrated that the cumulative size distribution of the LACs is governed by the exponent D of the power law. The original finding by Mishima et al. (1999) was based on a 2D CT image, but now, the power-law exponent D for LACs’ size distribution can be 3D calculated as shown in Figure 3. Following lung segmentation, each LAC is identified by 3D connecting neighboring LAA pixels, and the cumulative number of LACs *N(x)* larger than a given LAC size of x is counted. When the size *x* and *N(x)* can be fitted with a straight line on the log-log plot, the power-law exponent D can be obtained as the negative slope of the line. As shown in Figure 3, the progression of COPD leads to relatively larger LACs, which results in a flattening of the distribution and a smaller value of D.

Similar to the power-law exponent D for the size distribution of airspaces on histology, the power-law exponent D for the size distribution of LACs is not equal to the FD of the shape of LACs. Nonetheless, Mishima et al. (1999) further showed that a reduction in D is sensitive to capture parenchymal destruction in early-stage COPD, even when LAA% is not changed. In contrast, lung hyperinflation without parenchymal destruction in asthmatics is associated with an increase in LAA% without a change in D (Mitsunobu et al., 2003). A combination of the power-law exponent D and LAA% increased the accuracy of estimation of the pathological emphysema severity compared to the single-use of LAA% (Gietema et al., 2011) and accounted for sex differences in emphysema distribution (Camp et al., 2009). From the clinical perspective, the use of D has improved the predictive power for outcomes after a lung reduction surgery (Coxson et al., 2003) and a long-term prognosis (Hwang et al., 2019). In addition, D has also revealed relationships between exacerbation and emphysema progression (Tanabe et al., 2011) and between continuous smoking and spatially heterogeneous emphysema progression (Tanabe et al., 2012). Further, Shimizu et al. (2020) recently reported that while LAV% predicts lung function decline and 10-year mortality, the exponent D predicts shorter time to exacerbation in patients with COPD. This supports the notion that the power-law exponent D reflects a distinct clinical status that the more standard emphysema index LAV% does not include. Another advantage of using D over LAV% is the robustness against variations in the inspiratory level in repeated scans to detect longitudinal emphysema progression. Indeed, studies by Tanabe et al. (2011, 2012) demonstrated that a change in CT-based total lung volume between two scans is correlated with the change in LAV% but not with the exponent D. Hwang et al. (2016) also compared the values of D from inspiratory and expiratory CT and found that D did not differ between the two breath-hold levels. We, thus, propose that due to its robustness, the power-law exponent D is a more suitable biomarker than LAV%.

### Loss of the Power-Law for Size Distribution of Low Attenuation Regions on CT

**Figure 4:**
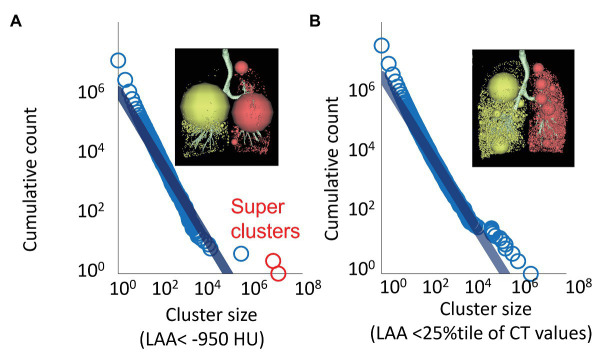
Superclusters not following the single power-law distribution and an alternative power-law analysis using relatively lower attenuation clusters on CT. **(A)** LACs were defined as 3D connecting voxels <−950 HU on CT. A log-log plot for cluster size and cumulative count of LACs larger than a given size showed that two very large clusters (red circles, superclusters) did not follow the power-law distribution. **(B)** Relatively lower attenuation clusters were defined using a threshold of the 25th percentile of the distribution of CT numbers instead of a fixed threshold to define emphysema, such as −950 HU.

Since the original discovery by Mishima et al. (1999) of the power-law behavior in the size distribution of LACs in COPD, countless studies have invoked the power-law exponent D based on the assumption that the power-law should be maintained in any parenchymal destruction in smokers. However, this concept was challenged by a recent study by Mondoñedo et al. (2019) who showed that as emphysema progresses, extremely large LACs appeared when the lung CT was analyzed in 3D (Figure 4). The authors further showed that the size of these extremely large LACs deviated from the expected power-law size distribution of the other LACs, and called them “superclusters.” On the log-log plot, the superclusters correspond to outliers to the fitted power-law distribution that are mathematically defined as LACs with volumes of at least 100 ml and squared residuals >0.5. It was found that the supercluster emerged between LAA% of 15 and 30% with a probability approaching one. Interestingly, however, by excluding the superclusters, the remaining LACs were found to follow a power-law size distribution as exemplified in Figure 4. Moreover, since the volume of the superclusters was closely associated with lung function in nine patients with COPD, it was argued that the supercluster was also driving the progression of the disease.

Tobino et al. (2017) compared the power law for the size distribution of LACs between COPD and other cystic lung diseases, including lymphangioleiomyomatosis (LAM) and Birt-Hogg-Dube syndrome (BHDS) based on 2D CT images. In that study, the power-law size distribution of LACs was maintained in more than 90% of CT images of patients with COPD and LAM, whereas the power-law was preserved in 63% of CT images in BHDS. Moreover, to enhance the robustness of the power-law analysis of low attenuation regions in COPD, Tobino et al. (2017) proposed that instead of a fixed threshold to define emphysema, such as −950 HU, the 15th, 25th, and 35th percentiles of the distribution of CT values can be used to define relatively lower attenuation regions and calculate the power-law exponent D (D'15, D'25, and D'35) of the corresponding size distribution. The authors found that compared to the conventional exponent D (Figure 4), D'15, D'25, and D'35 are more robust against variations in inspiratory level during CT scans. Furthermore, D'25, but not the conventional exponent D, D'15 or D'35, sensitively detects emphysema progression in current smokers, while, D'25, D'35, and the conventional exponent D had similar associations with airflow limitation and diffusing capacity. Collectively, the results of these recent reports warrant future investigations regarding the maintenance and loss of the power-law behavior in the size distribution of CT-based emphysema lesions during the progression of COPD.

### Simulation Studies

The discovery of the power law governing the size distribution of emphysematous clusters on CT has yielded insights into the pathogenesis of emphysema and COPD. Simulation has played an important role in this context. For example, Suki et al. (2003) constructed a spring network model and demonstrated that local enhancement of mechanical forces due to rupture of alveolar septum leads to further rupture and the iteration of this process creates spatially heterogeneous parenchymal destruction. Emphysema due to mechanical force-based destruction is thus characterized by a few large emphysematous clusters surrounded by many small emphysematous clusters on CT images, which not only results in a power-law distribution but also reduces the power-law exponent D as the rupture process proceeds.

Moreover, a longitudinal study found a decrease in the exponent D based on 2D CT images and an increase in LAA% in patients who experienced exacerbations during follow-up supported by model simulations using the baseline CT (Tanabe et al., 2011). In that simulation study, the authors converted non-emphysema pixels into emphysema pixels based on several pre-defined rules. Consequently, it was found that when selecting non-emphysema pixel randomly, LAA% was increased, but D did not decrease. In contrast, when selecting non-emphysema pixels separating regions of pre-existing LAA pixels, a new emphysema-pixel caused coalescence of pre-existing emphysematous clusters and induced both a decrease in the exponent D and an increase in LAA%, in agreement with the actual changes in D and LAA% in patients who experienced exacerbations. These findings suggest that exacerbations-induced coalescence of emphysematous clusters made pre-existing clusters larger, which supports the disruptive role of mechanical forces described above. Furthermore, these results also prove that D and LAA% do not always change simultaneously, which may explain why LAA% and D have different predictive roles in the long-term prognosis of COPD (Shimizu et al., 2020).

Subsequently, Tanabe et al. (2012) investigated the spatial pattern of emphysema progression induced by continuous smoking by combining the power-law analysis of LACs on CT with simulations. They found that smoking-induced emphysema progression is not uniform in the lung. New emphysematous lesions developed in local regions that were surrounded by pre-existing established emphysema clusters, leading to the spatially heterogeneous emphysema progression. Regarding the supercluster formation in COPD, Mondoñedo et al. (2019) introduced personalized computational modeling and showed that the mechanical forced-induced expansion of LACs and the coalescence of two neighboring LACs may cause the emergence of superclusters. This concept is consistent with a previous study by Bhatt et al. (2017) who used non-rigid registration of paired inspiratory and expiratory CTs in a large longitudinal observational study. They calculated the Jacobian determinant using the displacement field that was obtained in the process of non-rigid registration of inspiratory and expiratory CTs. The Jacobian determinant is an index for local lung expansion and contraction with respiration. The Jacobian determinant was calculated in normal regions adjacent to emphysema lesions. The results suggested that emphysematous regions had a greater Jacobian determinant than the other regions presumably due to abnormal mechanical stretch during breathing. Based on these findings, it was proposed that the extent of volume change in such regions could predict future lung function decline. This finding then suggests that normal regions are not uniformly damaged in the process of emphysema progression and that regions with normal CT density suffering from non-physiological mechanical stresses of cyclic volume change during breathing are vulnerable to further alveolar destruction.

In a study using an elastase-induced murine model of COPD, Sato et al. (2015) showed that the proteolytic injury and mechanical failure after elastase administration propagate beyond the initial distribution of elastase. Taken together with the simulation results discussed above, the following scenario emerges. Once an initial destruction of alveoli happens in the early stage of the disease, emphysema expands from this injury site by mechanical forces rupturing first nearby alveolar walls, followed by coalescence of the relatively larger neighboring emphysematous regions creating a power-law distribution of LACs. Eventually, the positive feedback between rupture and increased mechanical forces necessarily leads to the emergence of the supercluster. The effect of the giant supercluster is to allow non-physiological mechanical forces due to breathing to operate in its own neighborhood. Finally, any small additional enzymatic injury in the supercluster neighborhood weakens septal walls, and the non-physiologic forces will rupture the tissue so that the regions are absorbed into the ever-growing supercluster, which in turn is seen at the macroscopic scale as a breakdown of the power law of LACs.

## Airway Disease

### Self-Similarity and Fractal of Airway Tree

Airway trees also exhibit the fractal properties in human and animals (Horsfield, 1990; Weibel, 1991; Boser et al., 2005; Glenny, 2011; Glenny et al., 2020). An airway branch generally divides into two daughter branches. The ratio of length to diameter for each generation is reported as consistent using excised and fixed lungs (Weibel, 1991, 2009). In addition, the diameter ratio of daughter to mother branch is ~0.86, whereas the length ratio of daughter to mother branch is ~0.62 (Horsfield, 1990). The fact that these ratios are nearly independent of airway generation suggests that the branching pattern of the airway tree is characterized by self-similarity (Weibel, 1991; Glenny, 2011), and consequently, the structure of the airway tree has fractal properties. This conceptual framework has been applied to develop mathematical modeling of airway structure and various simulations in silico. For example, Nelson and Manchester (1988) suggested that the FD reflects tortuosity of biological trees. Moreover, Glenny et al. (2020) compared the FD of airway trees in different mouse strains and showed that fractal geometry is genetically coded.

### Airway Fractal Dimension and the Power-Law Exponent D on CT

**Figure 5:**
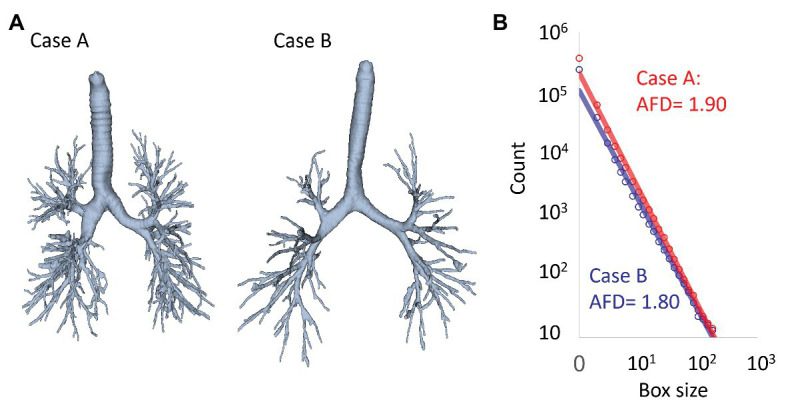
Fractal analysis of airway trees on CT based on the box-counting method. **(A)** Examples of segmented airway trees in two cases. **(B)** Using the box-counting method, the number of boxes that included the airway tree was counted. This counting was repeated for different box sizes. The count of boxes including the airway trees was plotted on a double logarithmic graph and the airway FD (AFD) was calculated as the negative slope of the regression lines (AFD = 1.90 and 1.80 for cases A and B).

The fractal properties of airway trees were also demonstrated by quantitative CT analyses (Bodduluri et al., 2018; Van de Moortele et al., 2018). A technical advancement in recent CT analysis software has made it possible to segment the airway tree automatically and more precisely up to the sixth generation airways (Van de Moortele et al., 2018). The fractal geometry of the segmented airway tree can then be analyzed using the 3D box-counting method (Bodduluri et al., 2018; Van de Moortele et al., 2018, 2019). A stack of CT images including the entire airway tree was 3D divided into cubic boxes of linear size *x*. The number of cubic boxes that include a part of the airway tree *N(x)* is counted for a given box size *x*. Then, the analysis is repeated for different values of *x* and *N(x)* that are plotted on a double logarithmic scale, and airway FD (AFD) is calculated as the negative slope of the regression line as shown in Figure 5.

Another method to apply the fractal concept to characterize the airway tree was introduced by Kitaoka and Suki (1997). The authors used data of human tracheobronchial geometry that was obtained from bronchial casts in two human lungs by Raabe et al. (1976) and showed that the cumulative distribution of the internal lumen diameter of airway branches is governed by a power law. This concept was recently reproduced by Kinose et al. (2020) who segmented airway tree from inspiratory CT scans and partitioned the whole tree into branches in 162 smokers with and without COPD. They measured the minimal inner CSA (iCSA) of airway branches and counted the cumulative number of airway branches *N(x)* having iCSA ≥ *x*. When the minimal lumen size *x* and *N(x)* were plotted on a double logarithmic scale, the power-law exponent D of iCSA was obtained as the negative slope of the regression line.

### Airway Fractal Dimension in Healthy Lungs

Airway fractal dimension in healthy smokers was first measured on 2D images of bronchial airway casts (Boser et al., 2005), and the mean AFD (1.83) in healthy subjects was higher than in those with fatal asthma (1.72) and in those with nonfatal asthma (1.76). On the 3D volumetric CT, Van de Moortele et al. (2018) reported that the mean AFD on inspiratory CT in 36 healthy lifelong nonsmokers with normal lung function (mean age 64) was 1.89. Meanwhile, Bodduluri et al. (2018) reported that the mean AFD in 105 healthy lifelong nonsmokers was 1.56, which was higher than that of smokers at risk (1.52) and subjects with COPD (1.45–1.50). Although both the reports applied the box-counting method to the 3D segmented airway tree, the values of the AFD in healthy subjects differed substantially in the two studies. We speculate that methods to segment and process airway trees and the box-counting methods implemented in different software might yield different outputs. Therefore, the methods to calculate AFD on volumetric CT should be standardized to increase the reliability of AFD in clinical applications. More studies are also necessary to understand how age, sex, and height affects AFD in healthy subjects.

### Airway Fractal Dimension and the Power-Law Analysis in COPD

Regarding airway tree morphology in COPD, Van de Moortele et al. (2019) showed that the central airway tree in subjects to airflow limitation is characterized by smaller diameters, smaller child-to-parent diameter ratios, larger length-to-diameter ratios, and a smaller AFD compared to healthy subjects. They also showed that a smaller AFD is associated with greater longitudinal decline in lung function over 5 years. Bodduluri et al. (2018) also found that a decrease in AFD is associated statistically significantly with airflow limitation on spirometry, quality of life, and 6-min walk distance independent of airway wall thickness and emphysema on the cross-sectional data. Importantly, a decrease in AFD at the baseline-study visit of the 5-year longitudinal cohort is an independent predictor for future exacerbations, lung function decline, and mortality in patients with COPD (Bodduluri et al., 2018). The authors performed simulations and found that AFD reflects the loss of airways which the conventional CT index of airway dimension, Pi10 (square root of the wall area of a hypothetical airway with a lumen perimeter of 10 mm), does not capture.

Interestingly, Kinose et al. (2020) showed that while both the power-law exponent D of iCSA and the AFD were correlated with airflow limitation, these indexes were weakly correlated with each other and proposed that the AFD based on box-counting of the airway tree and D of iCSA reflect different structural features. The AFD is related to the space-filling capacity of the entire tree in 3D whereas the power-law exponent D of the internal diameters characterizes the relative frequency of finding diameters larger than *x*, independent of their spatial arrangement in 3D. Furthermore, the authors also suggested that D of iCSA is influenced by the diameters of airway branches, but the AFD reflects the length and the number of branches rather than their diameter. While airways <2 mm in diameter are considered a main pathological site that a current multi-detector CT (MDCT) technique cannot resolve, a recently-introduced ultra-high-resolution CT may allow for direct evaluation of these airways (Tanabe et al., 2018b, 2019). Moreover, based on a recent MDCT report showing that the mismatch between central airway size and lung, called dysanapsis, is a main determinant of lung function and is closely associated with future incidence of COPD (Smith et al., 2020), the fractal property of the central airway tree may have an independent impact of clinical outcomes on COPD. Therefore, more efforts should be made to uncover distinct clinical values of these two fractal measurements.

## Vascular Diseases

Fractal-like structural properties have also been evaluated in the vasculature based on the idea of space-filling. The explanation could be found in the fractal-like architecture of the hierarchical branching vascular networks that distribute resources within organisms. The presence of fractal property in the vascular system can be closely associated with a reduction in the resistance to blood flow, which is also found in the fractal property of the leaf veins (Glenny and Robertson, 1990).

Studies in the 1990s evaluated the fractal property in the vasculature using contrast-enhanced CT images, but still little is known about the fractal property of the vasculature in diseased lungs, such as COPD. Haitao et al. (2011) reported that unlike emphysematous diseases, the FD of the pulmonary vessel structure (VFD) may be increased during the progression of pulmonary hypertension, whereas Helmberger et al. (2014) showed a positive correlation between the VFD and arterial oxygen saturation.

Very recently, Essay and Maina (2020) reported that the FD was 2.714 for the bronchial airway tree, 2.882 for the pulmonary arterial tree, and 2.334 for the pulmonary venous tree using latex rubber cast models. They also suggested that the human lung functionally complies with the Hess-Murray law or “the principle of minimum work.” The interrelationship between bronchial tree fractality and blood vessel fractality should be further investigated for better understanding of the pathophysiological nature of diseased lungs. Several studies using micro-CT have shown that the number of the terminal bronchioles decreases and that the cross-sectional lumen area of identifiable terminal bronchioles are reduced in COPD with both mild and severe airflow limitation (McDonough et al., 2011; Koo et al., 2018; Tanabe et al., 2018c). Since tissue oxygenation of peripheral airway trees are supplied by peripheral vasculature, there should be a loss of vasculature in lungs with COPD. We speculate that the loss of the peripheral small airways can be associated with the regions where the exponent D of the size distribution of the vascular diameters holds decreases while the VFD may remain unchanged. These predictions warrant further experiments.

## Discussion

In this review, we mainly focused on literature regarding the power-law and fractal analyses of the lung with COPD; however, we also made comparisons to asthma, LAM, and BHDS. Table 1 summarizes CT studies using the power-law and fractal analyses in patients with COPD. In reflection of the state of the current literature, more volume of content for emphysema was included in this review compared to airway disease and vessel abnormality. However, the power-law and fractal analyses of airway and vessel structures are also promising and upcoming research areas. Table 1 also shows that the range of exponent D for low attenuation regions and AFD vary among studies. The inconsistency might be due to variabilities in conditions of CT scanning such as reconstruction algorithms, different segmentation methods of airway and parenchyma, and the algorithms to calculate D and AFD. Segmentation of each target structure is required to perform these analyses, but this task, especially airway and vascular segmentations, is laborious for many clinicians in daily clinical practice. Moreover, to complete the power-law analysis for the size distribution of emphysematous low attenuation regions and airway branches, further computation is required to identify LACs from emphysema analysis and to partition the airway tree into branches for airway analysis. To overcome these current limitations and further characterize the fractal nature of morphology of COPD lungs, we recommend from a technical perspective that future studies attempt to standardize the methods and establish an automatic workflow to make the calculation of D and FD reproducible and available to attending physicians and radiologists. It would be important that scientists make the algorithm publicly available. From a pathophysiological perspective, we also recommend that studies investigate the mechanism by which the emergence in super emphysematous clusters induces a loss of the fractal property of the size distribution of emphysematous clusters, as well as by which AFD and D of iCSA change over time, specifically in different COPD phenotypes.

**Table 1:** Summary of CT studies regarding the power-law and fractal analysis of lung structure and COPD outcomes.

Authors	Target of the power-law and fractal analysis	Outcomes
**Parenchyma**		
Mishima et al., 1999	LAA size distribution	Detection of early COPD (exponent D: healthy 1.68, COPD with mild emphysema 1.38)
Coxson et al., 2003	LAA size distribution	LVRS response (exponent D: 0.56)
Gietema et al., 2011	LAA size distribution	Association with visual emphysema assessment (exponent D: panacinar emphysema 1.63, CLE 1.99, PSE 2.12)
Yuan et al., 2010	LAA size distribution	Estimate pathological emphysema (exponent D range: 0.1–2.5)
Camp et al., 2009	LAA size distribution	Sex difference in emphysema (exponent D: male 1.65, female 1.67)
Tanabe et al., 2011	LAA size distribution	Exacerbation and emphysema progression (exponent D: exacerbator 1.29, non-exacerbator 1.45)
Tanabe et al., 2012	LAA size distribution	Spatial pattern of smoking-induced emphysema progression (exponent D: former 1.83, current smokers 1.80)
Hwang et al., 2019	LAA size distribution	Long-term mortality (exponent D: 0.43)
Tanabe et al., 2018a	Relatively lower attenuation area size distribution	Sensitive detection of emphysema progression in current smoker (exponent D: former 1.25 current smokers 1.30)
Tobino et al., 2017	LAA size distribution	Differential diagnosis of COPD from other cystic lung diseases (exponent D not shown)
Mondoñedo et al., 2019	LAA size distribution	Super emphysema cluster emergence in disease progression (exponent D: control 1.63, COPD 1.30)
Shimizu et al., 2020	LAA size distribution	Future exacerbation (exponent D: 1.50)
**Airway**		
Bodduluri et al., 2018	Airway tree (box-counting)	Lung function decline, future exacerbation, and mortality (AFD: healthy 1.56, non-COPD smokers 1.52, COPD 1.45–1.50)
Kinose et al., 2020	Airway lumen size distribution	Longitudinal change in BMI (mean or range of exponent D not shown)
Van de Moortele et al., 2019	Airway tree (box-counting)	Airway tree morphology in healthy person (AFD: 1.89)
Van de Moortele et al., 2019	Airway tree (box-counting)	Lung function decline

LAA, low attenuation area on CT. LVRS, lung volume reduction surgery.

Since emphysema subtypes, including centrilobular, paraseptal, and panlobular emphysema (Lynch et al., 2015), show distinct clinical and pathological features (Kim et al., 1991; Saetta et al., 1994; Tanabe et al., 2017, 2020), future studies should also determine the fractal nature of each emphysema subtype. Moreover, in addition to the power-law exponent D of emphysema, a radiological finding of small airway disease on registered inspiratory and expiratory CT could be a CT-based biomarker of early-stage COPD (Galban et al., 2012; Bhatt et al., 2017; Vasilescu et al., 2019). Therefore, the relationship between the exponent D of emphysema and the radiographic evidence of small airway disease warrants further investigation.

The power-law and fractal concepts have been calculated for each component of lung structure. However, it is well known that airway-parenchymal interdependence substantially affects pulmonary function and airway disease is associated with the pathogenesis of emphysema. Therefore, an integrative approach, for example, that combined computational and mechanistic modeling with the power law for emphysema size distribution and the FD of airway tree, could open new directions in COPD research. These approaches, especially when combined with computer simulations, could lead to a better the understanding of the structural alterations in the early stages of COPD and help identify subjects at a high risk of severe COPD.

It should be also noted that in addition to fractal structures in the lung, a fractal pattern in ventilation heterogeneity is also known to exist (Altemeier et al., 2000). The fractal-like ventilation heterogeneity implies the presence of spatial clustering in which ventilation to a given region correlates with that of neighboring regions. Interestingly, the fractal properties of regional ventilation are similar to those of regional perfusion, implying strong ventilation-perfusion matching despite the large heterogeneity of both (Altemeier et al., 2000). Since non-invasive and more accessible imaging methods, such as MRI, has made it possible to quantitatively evaluate ventilation and perfusion (Hoffman et al., 2016; Voskrebenzev et al., 2018; Hatabu et al., 2020), the power-law and fractal analyses of the ventilation and perfusion mapping should be a promising research area to uncover the structure-function relationship in COPD. Taken together, the structural characterization and structure-function relationship of the lung and its various subsystems presented in this review may improve our understanding of the underlying mechanisms driving disease progression.

## Author Contributions

NT and SS made contributions to the design of this project and wrote the manuscript. BS and TH made contributions to the interpretation of the literatures and critically revised the manuscript for important intellectual content. All authors contributed to the article and approved the submitted version.

### Conflict of Interest

The authors declare that the research was conducted in the absence of any commercial or financial relationships that could be construed as a potential conflict of interest.

## References

[ref1] Adeloye D. Chua S. Lee C. Basquill C. Papana A. Theodoratou E.. (2015). Global and regional estimates of COPD prevalence: systematic review and meta-analysis. J. Glob. Health 5:020415. 10.7189/jogh.05-020415, PMID: 26755942 PMC4693508

[ref2] Altemeier W. A. McKinney S. Glenny R. W. (2000). Fractal nature of regional ventilation distribution. J. Appl. Physiol. 88, 1551–1557. 10.1152/jappl.2000.88.5.1551, PMID: 10797111

[ref3] Andersen M. P. Parham A. R. Waldrep J. C. McKenzie W. N. Dhand R. (2012). Alveolar fractal box dimension inversely correlates with mean linear intercept in mice with elastase-induced emphysema. Int. J. Chron. Obstruct. Pulmon. Dis. 7, 235–243. 10.2147/COPD.S26493, PMID: 22500123 PMC3324997

[ref4] Bhatt S. P. Bodduluri S. Hoffman E. A. Newell J. D. Jr. Sieren J. C. Dransfield M. T.. (2017). Computed tomography measure of lung at risk and lung function decline in chronic obstructive pulmonary disease. Am. J. Respir. Crit. Care Med. 196, 569–576. 10.1164/rccm.201701-0050OC, PMID: 28481639 PMC5620667

[ref5] Bodduluri S. Puliyakote A. S. K. Gerard S. E. Reinhardt J. M. Hoffman E. A. Newell J. D. Jr.. (2018). Airway fractal dimension predicts respiratory morbidity and mortality in COPD. J. Clin. Invest. 128, 5374–5382. 10.1172/JCI120693, PMID: 30256767 PMC6264725

[ref6] Boser S. R. Park H. Perry S. F. Menache M. G. Green F. H. (2005). Fractal geometry of airway remodeling in human asthma. Am. J. Respir. Crit. Care Med. 172, 817–823. 10.1164/rccm.200411-1463OC, PMID: 15976372

[ref7] Camp P. G. Coxson H. O. Levy R. D. Pillai S. G. Anderson W. Vestbo J.. (2009). Sex differences in emphysema and airway disease in smokers. Chest 136, 1480–1488. 10.1378/chest.09-0676, PMID: 19617404

[ref8] Caruthers S. D. Harris T. R. (1994). Effects of pulmonary blood flow on the fractal nature of flow heterogeneity in sheep lungs. J. Appl. Physiol. 77, 1474–1479. 10.1152/jappl.1994.77.3.1474, PMID: 7836155

[ref9] Coste F. Benlala I. Dournes G. Girodet P. O. Laurent F. Berger P. (2019). Assessing pulmonary hypertension in COPD. Is there a role for computed tomography? Int. J. Chron. Obstruct. Pulmon. Dis. 14, 2065–2079. 10.2147/COPD.S207363, PMID: 31564854 PMC6732516

[ref10] Coxson H. O. Nasute Fauerbach P. V. Storness-Bliss C. Muller N. L. Cogswell S. Dillard D. H.. (2008). Computed tomography assessment of lung volume changes after bronchial valve treatment. Eur. Respir. J. 32, 1443–1450. 10.1183/09031936.00056008, PMID: 18684848

[ref11] Coxson H. O. Whittall K. P. Nakano Y. Rogers R. M. Sciurba F. C. Keenan R. J.. (2003). Selection of patients for lung volume reduction surgery using a power law analysis of the computed tomographic scan. Thorax 58, 510–514. 10.1136/thorax.58.6.510, PMID: 12775863 PMC1746695

[ref12] Crawford J. W. Sleemant B. D. Young I. M. (1993). On the relation between number-size distributions and the fractal dimension of aggregates. J. Soil Sci. 44, 555–565.

[ref13] Essay M. Maina J. N. (2020). Fractal analysis of concurrently prepared latex rubber casts of the bronchial and vascular systems of the human lung. Open Biol. 10:190249. 10.1098/rsob.190249, PMID: 32634372 PMC7574555

[ref14] Galban C. J. Han M. K. Boes J. L. Chughtai K. A. Meyer C. R. Johnson T. D.. (2012). Computed tomography-based biomarker provides unique signature for diagnosis of COPD phenotypes and disease progression. Nat. Med. 18, 1711–1715. 10.1038/nm.2971, PMID: 23042237 PMC3493851

[ref15] Gevenois P. A. De Vuyst P. de Maertelaer V. Zanen J. Jacobovitz D.. (1996). Comparison of computed density and microscopic morphometry in pulmonary emphysema. Am. J. Respir. Crit. Care Med. 154, 187–192. 10.1164/ajrccm.154.1.8680679, PMID: 8680679

[ref16] Gietema H. A. Muller N. L. Fauerbach P. V. Sharma S. Edwards L. D. Camp P. G.. (2011). Quantifying the extent of emphysema: factors associated with radiologists’ estimations and quantitative indices of emphysema severity using the ECLIPSE cohort. Acad. Radiol. 18, 661–671. 10.1016/j.acra.2011.01.011, PMID: 21393027

[ref17] Glenny R. W. (2011). Emergence of matched airway and vascular trees from fractal rules. J. Appl. Physiol. 110, 1119–1129. 10.1152/japplphysiol.01293.2010, PMID: 21164156

[ref18] Glenny R. W. Krueger M. Bauer C. Beichel R. R. (2020). The fractal geometry of bronchial trees differs by strain in mice. J. Appl. Physiol. 128, 362–367. 10.1152/japplphysiol.00838.2019, PMID: 31917627 PMC7052590

[ref19] Glenny R. W. Robertson H. T. (1990). Fractal properties of pulmonary blood flow: characterization of spatial heterogeneity. J. Appl. Physiol. 69, 532–545. 10.1152/jappl.1990.69.2.532, PMID: 2228863

[ref20] Gould G. A. Macnee W. McLean A. Warren P. M. Redpath A. Best J. J.. (1988). CT measurements of lung density in life can quantitate distal airspace enlargement—an essential defining feature of human emphysema. Am. Rev. Respir. Dis. 137, 380–392. 10.1164/ajrccm/137.2.380, PMID: 3341629

[ref21] Grydeland T. B. Dirksen A. Coxson H. O. Eagan T. M. Thorsen E. Pillai S. G.. (2010). Quantitative computed tomography measures of emphysema and airway wall thickness are related to respiratory symptoms. Am. J. Respir. Crit. Care Med. 181, 353–359. 10.1164/rccm.200907-1008OC, PMID: 19926869

[ref22] Haitao S. Ning L. Lijun G. Fei G. Cheng L. (2011). Fractal dimension analysis of MDCT images for quantifying the morphological changes of the pulmonary artery tree in patients with pulmonary hypertension. Korean J. Radiol. 12, 289–296. 10.3348/kjr.2011.12.3.289, PMID: 21603288 PMC3088846

[ref23] Han M. K. Kazerooni E. A. Lynch D. A. Liu L. X. Murray S. Curtis J. L.. (2011). Chronic obstructive pulmonary disease exacerbations in the COPDGene study: associated radiologic phenotypes. Radiology 261, 274–282. 10.1148/radiol.11110173, PMID: 21788524 PMC3184233

[ref24] Haruna A. Muro S. Nakano Y. Ohara T. Hoshino Y. Ogawa E.. (2010). CT scan findings of emphysema predict mortality in COPD. Chest 138, 635–640. 10.1378/chest.09-2836, PMID: 20382712

[ref25] Hatabu H. Ohno Y. Gefter W. B. Parraga G. Madore B. Lee K. S.. (2020). Expanding applications of pulmonary MRI in the clinical evaluation of lung disorders: Fleischner society position paper. Radiology 297, 286–301. 10.1148/radiol.2020201138, PMID: 32870136

[ref26] Helmberger M. Pienn M. Urschler M. Kullnig P. Stollberger R. Kovacs G.. (2014). Quantification of tortuosity and fractal dimension of the lung vessels in pulmonary hypertension patients. PLoS One 9:e87515. 10.1371/journal.pone.0087515, PMID: 24498123 PMC3909124

[ref27] Hoffman E. A. Lynch D. A. Barr R. G. Van Beek E. J. Parraga G. Investigators I. (2016). Pulmonary CT and MRI phenotypes that help explain chronic pulmonary obstruction disease pathophysiology and outcomes. J. Magn. Reson. Imaging 43, 544–557. 10.1002/jmri.25010, PMID: 26199216 PMC5207206

[ref28] Horsfield K. (1990). Diameters, generations, and orders of branches in the bronchial tree. J. Appl. Physiol. 68, 457–461. 10.1152/jappl.1990.68.2.457, PMID: 2318756

[ref29] Hwang J. Lee M. Lee S. M. Oh S. Y. Oh Y. M. Kim N.. (2016). A size-based emphysema severity index: robust to the breath-hold-level variations and correlated with clinical parameters. Int. J. Chron. Obstruct. Pulmon. Dis. 11, 1835–1841. 10.2147/COPD.S109846, PMID: 27536095 PMC4976821

[ref30] Hwang J. Oh Y. M. Lee M. Choi S. Seo J. B. Lee S. M.. (2019). Low morphometric complexity of emphysematous lesions predicts survival in chronic obstructive pulmonary disease patients. Eur. Radiol. 29, 176–185. 10.1007/s00330-018-5551-7, PMID: 29959456

[ref31] Kim W. D. Eidelman D. H. Izquierdo J. L. Ghezzo H. Saetta M. P. Cosio M. G. (1991). Centrilobular and panlobular emphysema in smokers. Two distinct morphologic and functional entities. Am. Rev. Respir. Dis. 144, 1385–1390. 10.1164/ajrccm/144.6.1385, PMID: 1741553

[ref32] Kinose D. Ogawa E. Kawashima S. Matsuo-Kashiwagi Y. Yukimura-Seto R. Yamazaki A.. (2020). An index of the fractal characteristic of an airway tree is associated with airflow limitations and future body mass index reduction in COPD patients. J. Appl. Physiol. 128, 1280–1286. 10.1152/japplphysiol.00461.2019, PMID: 32240020

[ref33] Kitaoka H. Suki B. (1997). Branching design of the bronchial tree based on a diameter-flow relationship. J. Appl. Physiol. 82, 968–976. 10.1152/jappl.1997.82.3.968, PMID: 9074989

[ref34] Koo H. K. Vasilescu D. M. Booth S. Hsieh A. Katsamenis O. L. Fishbane N.. (2018). Small airways disease in mild and moderate chronic obstructive pulmonary disease: a cross-sectional study. Lancet Respir. Med. 6, 591–602. 10.1016/S2213-2600(18)30196-6, PMID: 30072106

[ref35] Labaki W. W. Martinez C. H. Martinez F. J. Galban C. J. Ross B. D. Washko G. R.. (2017). The role of chest computed tomography in the evaluation and management of the patient with chronic obstructive pulmonary disease. Am. J. Respir. Crit. Care Med. 196, 1372–1379. 10.1164/rccm.201703-0451PP, PMID: 28661698 PMC5736976

[ref36] Lynch D. A. Al-Qaisi M. A. (2013). Quantitative computed tomography in chronic obstructive pulmonary disease. J. Thorac. Imaging 28, 284–290. 10.1097/RTI.0b013e318298733c, PMID: 23748651 PMC4161463

[ref37] Lynch D. A. Austin J. H. Hogg J. C. Grenier P. A. Kauczor H. U. Bankier A. A.. (2015). CT-definable subtypes of chronic obstructive pulmonary disease: a statement of the fleischner society. Radiology 277, 192–205. 10.1148/radiol.2015141579, PMID: 25961632 PMC4613878

[ref38] Madani A. Zanen J. de Maertelaer V. Gevenois P. A. (2006). Pulmonary emphysema: objective quantification at multi-detector row CT—comparison with macroscopic and microscopic morphometry. Radiology 238, 1036–1043. 10.1148/radiol.2382042196, PMID: 16424242

[ref39] Mandelbrot B. (1977). The fractal geometry of nature. New York: W.H. Freeman and Company.

[ref40] Matsuoka S. Washko G. R. Yamashiro T. Estepar R. S. Diaz A. Silverman E. K.. (2010). Pulmonary hypertension and computed tomography measurement of small pulmonary vessels in severe emphysema. Am. J. Respir. Crit. Care Med. 181, 218–225. 10.1164/rccm.200908-1189OC, PMID: 19875683 PMC2817812

[ref41] McDonough J. E. Yuan R. Suzuki M. Seyednejad N. Elliott W. M. Sanchez P. G.. (2011). Small-airway obstruction and emphysema in chronic obstructive pulmonary disease. N. Engl. J. Med. 365, 1567–1575. 10.1056/NEJMoa1106955, PMID: 22029978 PMC3238466

[ref42] Mishima M. Hirai T. Itoh H. Nakano Y. Sakai H. Muro S.. (1999). Complexity of terminal airspace geometry assessed by lung computed tomography in normal subjects and patients with chronic obstructive pulmonary disease. Proc. Natl. Acad. Sci. U. S. A. 96, 8829–8834. 10.1073/pnas.96.16.8829, PMID: 10430855 PMC17692

[ref43] Mitsunobu F. Ashida K. Hosaki Y. Tsugeno H. Okamoto M. Nishida K.. (2003). Complexity of terminal airspace geometry assessed by computed tomography in asthma. Am. J. Respir. Crit. Care Med. 167, 411–417. 10.1164/rccm.2112070, PMID: 12554627

[ref44] Mondoñedo J. R. Sato S. Oguma T. Muro S. Sonnenberg A. H. Zeldich D.. (2019). CT imaging-based low-attenuation super clusters in three dimensions and the progression of emphysema. Chest 155, 79–87. 10.1016/j.chest.2018.09.014, PMID: 30292758 PMC6344385

[ref45] Muller N. L. Staples C. A. Miller R. R. Abboud R. T. (1988). “Density mask”. An objective method to quantitate emphysema using computed tomography. Chest 94, 782–787. 10.1378/chest.94.4.782, PMID: 3168574

[ref46] Nelson T. R. Manchester D. K. (1988). Modeling of lung morphogenesis using fractal geometries. IEEE Trans. Med. Imaging 7, 321–327. 10.1109/42.14515, PMID: 18230485

[ref47] Nishimura M. Makita H. Nagai K. Konno S. Nasuhara Y. Hasegawa M.. (2012). Annual change in pulmonary function and clinical phenotype in chronic obstructive pulmonary disease. Am. J. Respir. Crit. Care Med. 185, 44–52. 10.1164/rccm.201106-0992OC, PMID: 22016444

[ref48] Parr D. G. Sevenoaks M. Deng C. Stoel B. C. Stockley R. A. (2008). Detection of emphysema progression in alpha 1-antitrypsin deficiency using CT densitometry; methodological advances. Respir. Res. 9:21. 10.1186/1465-9921-9-21, PMID: 18271964 PMC2287169

[ref49] Parr D. G. Stoel B. C. Stolk J. Stockley R. A. (2006). Validation of computed tomographic lung densitometry for monitoring emphysema in alpha1-antitrypsin deficiency. Thorax 61, 485–490. 10.1136/thx.2005.054890, PMID: 16537666 PMC2111224

[ref50] Raabe O. G. Lovelace Foundation for Medical Education and Research National Institute of Environmental Health Sciences United States. Energy Research and Development Administration (1976). Tracheobronchial geometry: human, dog, rat, hamster--a compilation of selected data from the project respiratory tract deposition models. Washington: U.S. Energy Research and Development Administration, Division of Biomedical and Environmental Research: For sale by the Supt. of Docs., U.S. Govt. Print. Off.

[ref51] Saetta M. Kim W. D. Izquierdo J. L. Ghezzo H. Cosio M. G. (1994). Extent of centrilobular and panacinar emphysema in smokers’ lungs: pathological and mechanical implications. Eur. Respir. J. 7, 664–671. 10.1183/09031936.94.07040664, PMID: 8005246

[ref52] Sato S. Bartolak-Suki E. Parameswaran H. Hamakawa H. Suki B. (2015). Scale dependence of structure-function relationship in the emphysematous mouse lung. Front. Physiol. 6:146. 10.3389/fphys.2015.00146, PMID: 26029115 PMC4428081

[ref53] Sato A. Hirai T. Imura A. Kita N. Iwano A. Muro S.. (2007). Morphological mechanism of the development of pulmonary emphysema in klotho mice. Proc. Natl. Acad. Sci. U. S. A. 104, 2361–2365. 10.1073/pnas.0607882104, PMID: 17284608 PMC1892918

[ref54] Schroeder J. D. McKenzie A. S. Zach J. A. Wilson C. G. Curran-Everett D. Stinson D. S.. (2013). Relationships between airflow obstruction and quantitative CT measurements of emphysema, air trapping, and airways in subjects with and without chronic obstructive pulmonary disease. AJR Am. J. Roentgenol. 201, W460–W470. 10.2214/AJR.12.10102, PMID: 23971478 PMC4067052

[ref55] Shimizu K. Tanabe N. Tho N. V. Suzuki M. Makita H. Sato S.. (2020). Per cent low attenuation volume and fractal dimension of low attenuation clusters on CT predict different long-term outcomes in COPD. Thorax 75, 116–122. 10.1136/thoraxjnl-2019-213525, PMID: 31896733

[ref56] Smith B. M. Kirby M. Hoffman E. A. Kronmal R. A. Aaron S. D. Allen N. B.. (2020). Association of dysanapsis with chronic obstructive pulmonary disease among older adults. JAMA 323, 2268–2280. 10.1001/jama.2020.6918, PMID: 32515814 PMC7284296

[ref57] Suki B. Bates J. H. Frey U. (2011). Complexity and emergent phenomena. Compr. Physiol. 1, 995–1029. 10.1002/cphy.c100022, PMID: 23737210

[ref58] Suki B. Lutchen K. R. Ingenito E. P. (2003). On the progressive nature of emphysema: roles of proteases, inflammation, and mechanical forces. Am. J. Respir. Crit. Care Med. 168, 516–521. 10.1164/rccm.200208-908PP, PMID: 12941655

[ref59] Tanabe N. Muro S. Hirai T. Oguma T. Terada K. Marumo S.. (2011). Impact of exacerbations on emphysema progression in chronic obstructive pulmonary disease. Am. J. Respir. Crit. Care Med. 183, 1653–1659. 10.1164/rccm.201009-1535OC, PMID: 21471102

[ref60] Tanabe N. Muro S. Sato S. Oguma T. Sato A. Hirai T. (2018a). Fractal analysis of low attenuation clusters on computed tomography in chronic obstructive pulmonary disease. BMC Pulm. Med. 18:144. 10.1186/s12890-018-0714-5, PMID: 30157833 PMC6116481

[ref61] Tanabe N. Muro S. Sato S. Tanaka S. Oguma T. Kiyokawa H.. (2012). Longitudinal study of spatially heterogeneous emphysema progression in current smokers with chronic obstructive pulmonary disease. PLoS One 7:e44993. 10.1371/journal.pone.0044993, PMID: 23028728 PMC3445600

[ref62] Tanabe N. Oguma T. Sato S. Kubo T. Kozawa S. Shima H.. (2018b). Quantitative measurement of airway dimensions using ultra-high resolution computed tomography. Respir. Investig. 56, 489–496. 10.1016/j.resinv.2018.07.008, PMID: 30392536

[ref63] Tanabe N. Shima H. Sato S. Oguma T. Kubo T. Kozawa S.. (2019). Direct evaluation of peripheral airways using ultra-high-resolution CT in chronic obstructive pulmonary disease. Eur. J. Radiol. 120:108687. 10.1016/j.ejrad.2019.108687, PMID: 31574362

[ref64] Tanabe N. Vasilescu D. M. Hague C. J. Ikezoe K. Murphy D. T. Murphy D. T. (2020). Pathological comparisons of paraseptal and centrilobular emphysema in chronic obstructive pulmonary disease. Am. J. Respir. Crit. Care Med. 202, 803–811. 10.1164/rccm.201912-2327OC 32485111

[ref65] Tanabe N. Vasilescu D. M. Kirby M. Coxson H. O. Verleden S. E. Vanaudenaerde B. M.. (2018c). Analysis of airway pathology in COPD using a combination of computed tomography, micro-computed tomography and histology. Eur. Respir. J. 51:1701245. 10.1183/13993003.01245-2017, PMID: 29444912 PMC6691959

[ref66] Tanabe N. Vasilescu D. M. McDonough J. E. Kinose D. Suzuki M. Cooper J. D.. (2017). Micro-computed tomography comparison of preterminal bronchioles in centrilobular and panlobular emphysema. Am. J. Respir. Crit. Care Med. 195, 630–638. 10.1164/rccm.201602-0278OC, PMID: 27611890 PMC5363975

[ref67] Tobino K. Hirai T. Johkoh T. Fujimoto K. Kawaguchi A. Tomiyama N.. (2017). Difference of the progression of pulmonary cysts assessed by computed tomography among COPD, lymphangioleiomyomatosis, and Birt-Hogg-Dube syndrome. PLoS One 12:e0188771. 10.1371/journal.pone.0188771, PMID: 29220357 PMC5722335

[ref68] Van De Moortele T. Goerke U. Wendt C. H. Coletti F. (2019). Airway morphology and inspiratory flow features in the early stages of chronic obstructive pulmonary disease. Clin. Biomech. 66, 60–65. 10.1016/j.clinbiomech.2017.11.005, PMID: 29169684 PMC5955793

[ref69] Van De Moortele T. Wendt C. H. Coletti F. (2018). Morphological and functional properties of the conducting human airways investigated by *in vivo* computed tomography and *in vitro* MRI. J. Appl. Physiol. 124, 400–413. 10.1152/japplphysiol.00490.2017, PMID: 29097628 PMC5867369

[ref70] Vasilescu D. M. Martinez F. J. Marchetti N. Galban C. J. Hatt C. Meldrum C. A.. (2019). Noninvasive imaging biomarker identifies small airway damage in severe chronic obstructive pulmonary disease. Am. J. Respir. Crit. Care Med. 200, 575–581. 10.1164/rccm.201811-2083OC, PMID: 30794432 PMC6727153

[ref71] Vestbo J. Edwards L. D. Scanlon P. D. Yates J. C. Agusti A. Bakke P.. (2011). Changes in forced expiratory volume in 1 second over time in COPD. N. Engl. J. Med. 365, 1184–1192. 10.1056/NEJMoa1105482, PMID: 21991892

[ref72] Voskrebenzev A. Gutberlet M. Klimes F. Kaireit T. F. Schonfeld C. Rotarmel A.. (2018). Feasibility of quantitative regional ventilation and perfusion mapping with phase-resolved functional lung (PREFUL) MRI in healthy volunteers and COPD, CTEPH, and CF patients. Magn. Reson. Med. 79, 2306–2314. 10.1002/mrm.26893, PMID: 28856715

[ref73] Weibel E. R. (1991). Fractal geometry: a design principle for living organisms. Am. J. Phys. 261, L361–L369. 10.1152/ajplung.1991.261.6.L361, PMID: 1767856

[ref74] Weibel E. R. (2009). What makes a good lung? Swiss Med. Wkly. 139, 375–386. PMID: 19629765 10.4414/smw.2009.12270

[ref75] Wilson D. O. Weissfeld J. L. Balkan A. Schragin J. G. Fuhrman C. R. Fisher S. N.. (2008). Association of radiographic emphysema and airflow obstruction with lung cancer. Am. J. Respir. Crit. Care Med. 178, 738–744. 10.1164/rccm.200803-435OC, PMID: 18565949 PMC2556456

[ref101] Yuan R. Nagao T. Pare P. D. Hogg J. C. Sin D. D. Elliott M. W. (2010). Quantification of lung surface area using computed tomography. Respir Res. 11:153. 21040527 10.1186/1465-9921-11-153 PMC2976969

